# Transition to Targeted Therapies Improved the Prognosis and Increased the Utilization of Medical Treatments among Patients with Synchronous Metastatic Renal Cell Cancer

**DOI:** 10.1155/2021/5237695

**Published:** 2021-08-12

**Authors:** Lauri Laru, Hanna Ronkainen, Markku H. Vaarala

**Affiliations:** Department of Surgery, Medical Research Center Oulu, Oulu University Hospital, University of Oulu, P.O. Box 21, 90029 OYS, Oulu, Finland

## Abstract

Since the introduction of targeted therapies (TTs) for metastatic renal cell cancer (mRCC) in 2005, a limited amount of epidemiological data on efficacy of modern drug therapies for synchronous mRCC has been published. We present a comprehensive nationwide cohort including all cases of primarily metastasized renal cell cancer among adults diagnosed between 2005 and 2010, based on data from the Finnish Cancer Registry and patient records from treating hospitals. Applied treatment protocols and survival outcomes were analyzed. A total of 977 patients were included in the analysis; 499 patients were diagnosed between 2005 and 2007 and 478 patients were diagnosed between 2008 and 2010. The median overall survival (OS) was 8.80 months (95% confidence interval (CI): 7.60–10.02). The median OS of the patients diagnosed at the latter era was significantly better (11.1; 95% CI: 8.8–13.4 vs. 7.0; 95% CI: 5.7–8.3 months, *p* ≤ 0.001). A total number of 524 (53.8%) patients received drug therapy. Altogether, TTs including tyrosine kinase inhibitors, mammalian target of rapamycin inhibitors (mTORi), and vascular endothelial growth factor inhibitor covered 331 (63.2%) of first-line treatments, whereas interferon and its combinations with chemotherapy were used for 186 (35.5%) patients. The median OS rates for TT and interferon as first-line therapy groups were 19.9 (16.9–22.8) and 14.9 (12.3–17.4) months, respectively. The OS for patients who did not receive drug therapy after cytoreductive nephrectomy was dismal. We found that the OS estimate of mRCC patients in Finland has improved since the introduction of tyrosine kinase inhibitors. However, the prognosis remains poor for frail, elderly patients with an impaired performance status.

## 1. Introduction

Approximately 20–30% of renal cell cancer (RCC) patients have distant metastases at the time of initial diagnosis [1], thus being diagnosed with synchronous metastatic renal cell carcinoma (mRCC). A limited amount of epidemiologic data on mRCC has been published, and although the safety and efficacy of modern drug therapies have been shown in randomized controlled trials, there is a scarcity of evidence on the effect of advances in medical therapy on the mRCC population. Population-based registry studies from Norway, Denmark, Sweden, Estonia, and Czech Republic [2–6] have been published during the last decade, and according to these studies, the prognosis remains relatively poor, as overall survival (OS) of only 9–14 months for the entire mRCC population is reported in these studies. Moreover, synchronous and metachronous metastases are shown to have different prognoses, with synchronous metastatic disease tending to be of a more aggressive phenotype [7, 8]. Whereas the role of cytoreductive nephrectomy remains controversial, clear advances in drug therapy have been made during the last 2 decades [9–11].

Until 2005, cytokine-based treatment with interferon alpha-2b (IFN-*α*), or less frequently interleukin-2, was considered the cornerstone of drug therapy but has been subsequentially replaced with targeted therapy (TT) such as vascular endothelial growth factor (VEGF) monoclonal antibodies and VEGF receptor tyrosine kinase inhibitors (TKIs). Since phase three trials showed the superiority of sunitinib compared to IFN-*α* with tolerable side effects, TKIs have rapidly become the standard of care in treatment-naïve clear cell mRCC, surpassing cytokine treatment [12, 13]. The efficacy of TKIs has been confirmed in multiple studies, showing improved OS times from 18.8 up to 52 months in selected patient populations [14, 15].

Lately, novel immunooncologic treatments with immune checkpoint inhibitors have shown promising results and are currently gaining a robust position as an option to TKIs in first-line treatment of mRCC [16]. However, owing to the substantial cost of checkpoint inhibitors and wide clinical experience and evidence regarding TKIs, sunitinib, pazopanib, and cabozantinib still remain as the frequently used first-line treatments in Finland.

In this study, we present the patient characteristics, applied treatment protocols, and outcomes of a nationwide cohort of 977 patients with synchronous mRCC diagnosed between 2005 and 2010. Furthermore, as a rapid transition in the standard drug treatment of these patients occurred from 2007, we aimed to investigate the impact of the diagnostic time period to the survival estimate, performing separate analysis for patients diagnosed in the cytokine (2005–2007) and targeted therapy era (2008–2010).

## 2. Materials and Methods

Data from all patients with synchronous mRCC or RCC with unknown metastatic status diagnosed between 2005 and 2010 were identified from the Finnish Cancer Registry, which includes all new cancer cases in Finland. Based on these data, patient records of 2,169 consecutively diagnosed patients were requested from the according hospitals. The following numbers of patients were excluded from the analysis: 410, 500, 20, and 57 patients were diagnosed outside the defined timeframe, had no evidence of metastasis at the time of diagnosis, were under 18 years of age, and had other cancers with advanced stage, respectively. Further, 31 posthumously diagnosed cases were excluded. Also, 166 patients with insufficient data on the time of diagnosis, end of surveillance, received treatments, or metastatic stage were ruled out. In addition, due to nonrenal cell cancer histology, 2 cases of poorly differentiated urothelial carcinoma, 3 neuroendocrine/small cell carcinomas, 1 malignant epithelioid angiomyolipoma, 1 Wilms' tumor, and 1 leiomyosarcoma were ruled out, resulting in a total number of 977 patients included in the final analysis. The following clinicopathologic variables were collected: sex, age at the time of diagnosis, primary cancer characteristics (T stage, Fuhrman grade, and histology), metastasis details (location of metastasis and number of metastatic sites), Eastern Cooperative Oncology Group (ECOG) performance status, laboratory results (serum hemoglobin and C-reactive protein (CRP)), nephrectomy status, and cause of death. T stage was reassigned according to the 2017 TNM classification [17] and ECOG performance status at the time of diagnosis was evaluated retrospectively by the author if not clearly specified in the patient records.

Treatment protocols, follow-up frequency, and modality were at the discretion of the treating physician. At least one dose of the drug therapy for RCC was required to include the patient in the analyses for drug therapies.

### 2.1. Statistical Analysis

The main outcome, OS, was defined as the time from diagnosis to death, otherwise censored at last follow-up contact. The survival distribution and median survival were assessed with Kaplan–Meier estimates. Significance was taken at *p* ≤ 0.05. Log-rank tests were used to test the influence of treatments on OS. Comparing baseline characteristics between diagnostic period groups, Pearson Chi-square test, Mann–Whitney *U* test, and Student's *t* test were used for categorical, ordinal, and continuous variables, respectively. Statistical analysis was performed using IBM SPSS version 26 (Chicago, IL, USA).

## 3. Results

### 3.1. Patient Characteristics and Biochemical and Pathological Features

Baseline characteristics are shown in Table 1. A total of 977 patients were included in the analysis: 564 (57.7%) male and 413 (42.3%) female. Mean age was 68.4 years (standard deviation (SD): 11.8 years). Mean follow-up was 22.8 months. From the 656 patients with available histological diagnosis, 584 (89.0%) presented with clear cell histology and 72 (11.0%) with other histology. Sarcomatoid changes were found in 56 (8.6%) of these patients. For 321 patients, histological diagnosis was not available. Patients identified with a local tumor stage: T1, 182 (20.3%); T2, 151 (16.9%); T3, 401 (44.8%); and T4, 162 (18.1%). Additionally, T staging was not reliably defined for 81 (8.3%) patients. Median primary tumor maximum diameter was 9.0 (range: 1.0–25.0) cm. Nephrectomy was performed for 518 patients. Owing to the retrospective nature of this study, serum calcium, neutrophil, and lactate dehydrogenase levels were not available for analysis for all patients; therefore, International Metastatic RCC Database Consortium (IMDC) or Memorial Sloan Kettering Cancer Center (MSKCC) risk criteria were not evaluated. However, serum hemoglobin and CRP levels were retrieved for most of the patients.

### 3.2. Drug Therapies for mRCC and Respective Outcomes

Median OS was 8.80 (95% confidence interval (CI): 7.60–10.02) months. A total number of 524 (53.8%) patients received drug therapy. In first-line treatment, sunitinib was the most frequently used and administered to 278 (53.1%) patients. IFN-*α* was the second most frequently used drug in a first-line setting (114 patients, 21.4%), followed by a combination of interferon and vinblastine (58 patients, 11.1%). All first-line treatments and respective median overall survivals are listed in Table 2. Altogether, TTs including tyrosine kinase inhibitors (TKIs: sunitinib, sorafenib, pazopanib, and regorafenib); mTORis (temsirolimus and everolimus); and VEGF inhibitor (VEGFi; bevacizumab) covered 331 (63.2%) of first-line treatments, whereas interferon and its combinations with chemotherapy (i.e., vinblastine and capecitabine) were used for 186 patients (35.5%). Other first-line drug therapies were single or a combination of cytotoxic chemotherapies.

### 3.3. Cytoreductive Nephrectomy among Patients without Drug Therapies for mRCC

**Figure 1:**
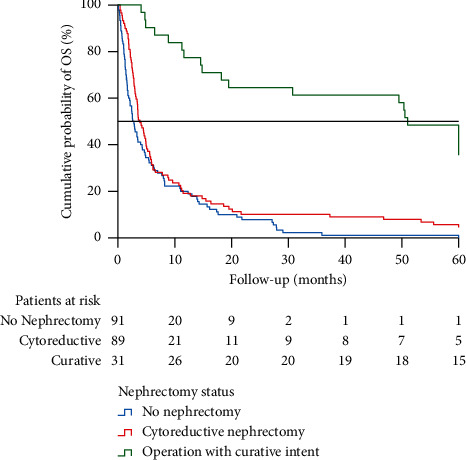
Kaplan–Meier analysis of overall survival in patients who did not receive drug therapies for histologically confirmed renal cell cancer. For this analysis, the follow-up time was limited to 60 months due to low number of patients surviving longer.

As cytoreductive nephrectomy was a recommended treatment during the study period, the effect of this major surgery on median OS was separately evaluated among patients who did not receive drug therapies for mRCC. Only patients with primary tumor histology available were analyzed. Cytoreductive nephrectomy, or nephrectomy and metastasectomy with curative intent, was performed for 89 and 31 patients who did not receive drug therapies for mRCC, respectively. Ninety patients were not treated with either nephrectomy or drug therapies. Though four patients were alive at the end of the follow-up after cytoreductive nephrectomy only, the median OS after cytoreductive nephrectomy was poor: 3.88 (95% CI 2.80–4.96) months (Figure 1). The OS for patients with no surgical or drug therapies for mRCC was 2.60 (95% CI 1.79–3.40) months. The dismal prognosis of cytoreductive nephrectomy is at least partly explained by the fact that 43 patients died during 90 days after nephrectomy.

### 3.4. Time of Diagnosis as a Prognostic Factor

**Figure 2:**
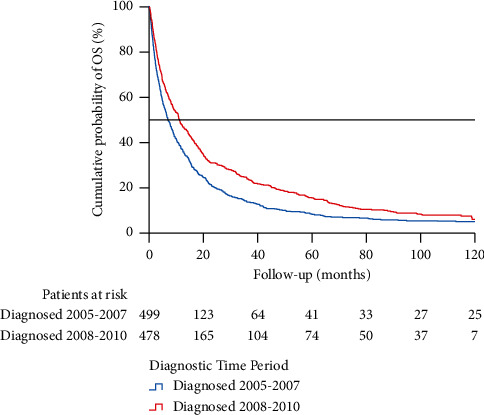
Kaplan–Meier analysis comparing overall survival of patients according to their diagnostic period (2005–2007 vs. 2008–2010).

As the paradigm shift from interferon to TTs in standard drug therapy followed the deployment of sunitinib in 2007, we chose to separately examine cases diagnosed between 2005 and 2007, compared to 2008–2010. A total of 499 patients were diagnosed between 2005 and 2007 and 478 patients between 2008 and 2010. The median OS of the patients diagnosed at the latter era was significantly better (11.1; 95% CI: 8.8–13.4 months vs. 7.0; 95% CI: 5.7–8.3 months, *p* ≤ 0.001) (Figure 2). The proportions of patients treated with interferon and TTs and those without drug therapies for mRCC, with respective OS rates, are presented in Table 3. The OS tended to improve among patients treated with TTs as first-line drug therapy.

## 4. Discussion

In this study, we present a nationwide real-life cohort including all clinically diagnosed primarily metastasized renal cell cancers among adults from a six-year period.

The estimated overall survival of all patients was 8.8 (95% CI 7.6–10.0) months. This correlates closely to results from a Norwegian population-based study, which reported a median OS of 9.0 (95% CI 7.9–10.1) months for all primary mRCC patients diagnosed between 2002 and 2011 [2]. A Swedish population-based study reported an OS of 12.4 (95% CI 11.3–13.8) for mRCC patients diagnosed between 2006 and 2008, but only 31% of these patients presented with metastatic disease at initial diagnosis, which makes the data not directly comparable to ours [4]. Median OS for mRCC patients receiving drug therapies in the Danish population increased from 11.5 to 17.2 months from 2006 to 2010, but OS for untreated patients remained at 3.0 months for the same period, which is identical to our results [3].

A significant proportion of cases represented a frail elderly population with reduced overall health status and limited tolerance of surgical treatment or drug therapies for mRCC. Although an age ≥75 years has not been proven as an independent prognostic factor for mRCC patients [18], it explains the relatively poor overall survival compared to various mRCC studies devoted to histologically confirmed clear cell tumors in patients with relatively good (ECOG 0–1) performance status. Cytoreductive nephrectomy as the only treatment resulted in no OS advantage. Almost half of the patients, who did not receive drug therapies for mRCC, deceased during 90 days after nephrectomy. However, single patients had excellent prognosis after cytoreductive nephrectomy only, which may be explained by spontaneous regression of the metastases [19–21], or incorrect radiological diagnosis of mRCC. Cytoreductive nephrectomy is major surgery especially in locally advanced cases, and the patients should fit well for surgery.

The OS observed in the first-line sunitinib group in our unselected patient population was 20.1; 95% CI: 15.7–24.5 months, which is comparable to median OS of 18.4 months reported in an expanded-access sunitinib trial published in 2009, including 7% patients with brain metastases, 13% with an ECOG performance status of 2 or worse, and 13% nonclear cell RCC, presenting a more diverse patient population [22]. In comparison, a median OS of 28.6 months was reported in a recent retrospective analysis including clear cell mRCC patients from all IMDC risk groups treated with first-line sunitinib, noting a prominent difference in the median OS of IMDC favorable (52.1, 95%CI: 43.4–61.2 months) vs. combined IMDC intermediate and poor (23.2, 95% CI: 21.0–25.8 months) groups [15].

Among the patients with a poorer performance status, histological diagnosis was also frequently missing. This resulted mostly from the fact that acquiring histology was not judged to be essential in cases with absence of effective surgical or drug therapy options for mRCC, due to the poor general health of the patient. However, a proportion of patients received drug therapies for mRCC despite lacking histological diagnosis (data not shown).

In the pivotal phase three sunitinib vs. interferon trial by Motzer et al. [12], the median progression-free survival was significantly longer in the sunitinib group compared to the interferon group (11 vs. 5 months, respectively). Subsequently, a significant OS benefit for the sunitinib group (26.4 vs. 21.8 months) compared to the IFN-*α* group was reported [13]. In our investigation, a similar trend of OS difference in sunitinib and IFN-*α* groups (20.1 and 14.5 months, respectively) was observed; however, this remains statistically insignificant.

We found that the overall survival prognosis of mRCC improved significantly during the observed period, which upholds the similar findings from other population-based studies [2–6]. We found a significant difference in the baseline factors ECOG and increased CRP value between diagnostic time groups in favour of the latter era, but no statistically significant difference in the number or location of metastatic sites or other baseline factors (Table 1). Thus, the data suggests that the overall treatment of the more recently diagnosed patient population has been more effective. The transition of primary first-line drug therapy from interferon-based regimes to TT and simultaneous improvement of OS can be observed in Table 3 and Figure 2, respectively.

The greatest strength of our study is that it is based on the data from the Finnish Cancer Registry, which receives a notification of every suspected or diagnosed cancer in Finland directly from the treating hospital [23]. Based on this information, complementary patient record acquisitions from the hospitals were made to form a comprehensive review of the available patient data, describing the national situation as accurately as possible. The patient records were thoroughly examined by the author, collecting all the planned information that was available. In addition to the effects of drug therapies in a large real-life cohort, this study brings forth valuable and reliable information on the natural history of synchronous mRCC, which is not a widely covered subject in the current epidemiologic literature.

However, our study has obvious limitations. It is a retrospective study, and there are some proportions of missing data. Particularly, laboratory results were not comprehensively available in many cases, probably because they were not included in the clinical practice at that time. We were not able to retrieve the original diagnostic imaging material for reevaluation but relied on the local radiology reports on assessing the diagnostic stage and radiologic progression or treatment response. In the present study, if ECOG performance status was not assessed and recorded at diagnosis, it was assigned retrospectively by the author, when judged possible based on the available information. It would be of great interest to evaluate the effect of novel immunooncological therapies among this patients population, but due to financial issues, the use of this treatment modality is just expanding in Finland at the moment. So the perfect time for this analysis is yet to come.

## 5. Conclusion

A rapid change of treatment of choice from cytokines to TT occurred in Finland between 2007 and 2008. This resulted in a significant improvement in OS of mRCC patients. Our findings suggest that cytoreductive nephrectomy should be performed only for patients not likely to waste away after surgery. A significant proportion of mRCC patients are diagnosed in a late symptomatic phase of the disease, and for such patients, the overall survival remains poor despite the advancements in cancer therapy.

## Figures and Tables

**Table 1:** Baseline characteristics of patient population according to diagnostic period.

Baseline characteristics	Number of patients (%)			*p*-value
	Diagnostic period		Total	
	2005–2007	2008–2010		
Total number of patients	499	478	977	

Gender				0.094
M	301 (60.3%)	263 (55.0%)	564 (57.7%)	
F	198 (39.7%)	215 (45.0%)	413 (42.3%)	
Mean age at diagnosis (years)	68.6	68.8	68.4	0.619

ECOG^*∗*^				**<0.001**
0	23 (5.5%)	40 (8.6%)	63 (7.2%)	
1	202 (48.6%)	233 (50.3%)	435 (49.5%)	
2	103 (24.8%)	124 (26.8%)	227 (25.8%)	
3	61 (14.7%)	59 (12.7%)	120 (13.7%)	
4	27 (6.5%)	7 (1.5%)	34 (3.9%)	

*T* stage^*∗*^				0.567
*T*1	95 (21.7%)	87 (19.0%)	182 (20.3%)	
*T*2	69 (15.8%)	82 (17.9%)	151 (16.9%)	
*T*3	191 (43.6%)	210 (45.9%)	401 (44.8%)	
*T*4	83 (18.9%)	79 (17.2%)	162 (18.1%)	

*N* stage				0.507
*N*0	305 (61.1%)	302 (63.2%)	607 (62.1%)	
*N*1	194 (38.9%)	176 (36.8%)	370 (37.9%)	

Number of metastatic sites				0.823
1	150 (30.1%)	135 (28.2%)	285 (29.2%)	
2	159 (31.9%)	156 (32.6%)	315 (32.2%)	
≥3	190 (38.1%)	187 (39.1%)	377 (38.6%)	

Metastatic sites				
Distant lymph nodes	112 (22.4%)	133 (27.8%)	245 (25.1%)	0.052
Lungs	297 (59.5%)	302 (63.2%)	599 (61.3%)	0.240
Bone	154 (30.9%)	159 (33.3%)	313 (32.0%)	0.421
Adrenal gland	101 (20.2%)	87 (18.2%)	188 (19.2%)	0.419
Liver	102 (20.4%)	96 (20.1%)	198 (20.3%)	0.890
Cerebral	30 (21.0%)	25 (24.0)	55 (22.3%)	0.568

Histology tumor type^*∗*^				0.800
Clear cell carcinoma	285 (89.3%)	299 (88.7%)	584 (89.0%)	
Other	34 (10.7%)	38 (11.3%)	72 (11.0%)	

Nephrectomy	246 (49.3%)	272 (56.9%)	518 (53.0%)	0.017
Hemoglobin < LLN^*∗*^	193 (60.5%)	207 (60.5%)	400 (60.5%)	0.995
CRP > ULN	211 (82.1%)	216 (74.7%)	427 (78.2%)	**0.038**

^*∗*^Histological diagnosis was missing for 318 patients: T stage for 78, hemoglobin for 313, CRP for 428, and ECOG status for 95 patients. The percentages in the second column were calculated only for the group of patients for whom data on these variables were available. Abbreviations: IQR = interquartile range; LLN = lower limit of normal; ULN = upper limit of normal.

**Table 2:** First-line drug therapies and median overall survival (OS) times with respective 95% confidence intervals (CIs).

Drug	No. of patients (%)	Median OS (95% CI) (months)
No drug therapy	453 (46.4%)	3.0 (2.6–3.4)
Sunitinib	278 (28.5%)	20.1 (15.7–24.5)
Interferon single	112 (11.5%)	14.5 (8.5–20.6)
Interferon + vinblastine	58 (5.9%)	15.6 (12.6–18.7)
Interferon + capecitabine	16 (1.6%)	13.4 (1.8–25.0)
Temsirolimus	16 (1.6%)	10.8 (8.5–13.1)
Sorafenib	15 (1.5%)	19.9 (0.0–44.5)
Pazopanib	7 (0.7%)	19.7 (16.0–23.5)
Bevacizumab	7 (0.7%)	15.4 (8.9–22.0)
Bevacizumab + interferon	3 (0.3%)	54.5 (4.1–104.8)
Regorafenib	3 (0.3%)	51.3 (0.0–125.9)
Vinblastine	2 (0.2%)	1.5
Everolimus	1 (0.1%)	8.5
Vinblastine + bevacizumab	1 (0.1%)	9.2
Etoposide	1 (0.1%)	3.3
Ifosfamide + doxorubicin	1 (0.1%)	18.3
Capecitabine	1 (0.1%)	6.4
Erlotinib	1 (0.1%)	10.3
Ifosfamide and mesna + adriamycin	1 (0.1%)	6.1
Overall	977 (100.0%)	8.7 (7.5–9.9)

**Table 3:** First-line drug therapies according to time of diagnosis. Number of patients and overall survival (OS) rates with 95% confidence intervals (CIs) are presented. Seven patients received only chemotherapy and were excluded from data presented in this table.

First-line drug therapy	Time of diagnosis				Total
	2005–2007 (*N* = 494)		2008–2010 (*N* = 476)		*N* = 970
	*n* (%)	OS (95% CI)	*n* (%)	OS (95% CI)	*n* (%)
No medical treatment	260 (52.1)	2.86 (2.22–3.50)	193 (40.4)	3.38 (2.77–4.00)	453 (46.4)
Interferon ± chemotherapy	169 (33.9)	15.3 (12.5–18.1)	17 (3.6)	13.2 (4.74–21.6)	186 (19.0)
TT	65 (13.0)	14.8 (11.9–17.6)	266 (55.6)	20.2 (16.3–24.1)	331 (33.9)

## Data Availability

Restrictions apply to the availability of these data, which were used under license for the current study, and so are not publicly available. Data are, however, available from the authors upon reasonable request.
